# A clinical radiomics nomogram preoperatively to predict ductal carcinoma in situ with microinvasion in women with biopsy-confirmed ductal carcinoma in situ: a preliminary study

**DOI:** 10.1186/s12880-023-01092-5

**Published:** 2023-09-07

**Authors:** Zhou Huang, Xue Chen, Nan Jiang, Su Hu, Chunhong Hu

**Affiliations:** 1 Department of Radiology, the First Affiliated Hospital of Soochow University, No. 899 Pinghai Road, Gusu District, Suzhou City, Jiangsu Province 215006 PR China; 2 Department of Radiology, the Affiliated Suzhou Hospital of Nanjing Medical University, Suzhou Municipal Hospital, Suzhou City, Jiangsu Province 215002 PR China

**Keywords:** Ductal carcinoma in situ, Ductal carcinoma in situ with microinvasive, MRI, Clinicopathologic, Radiomics, Upstage

## Abstract

**Purpose:**

To predict ductal carcinoma in situ with microinvasion (DCISMI) based on clinicopathologic, conventional breast magnetic resonance imaging (MRI), and dynamic contrast enhanced MRI (DCE-MRI) radiomics signatures in women with biopsy-confirmed ductal carcinoma in situ (DCIS).

**Methods:**

Eighty-six women with eighty-seven biopsy-proven DCIS who underwent preoperative MRI and underwent surgery were retrospectively identified. Clinicopathologic, conventional MRI, DCE-MRI radiomics, combine (based on conventional MRI and DCE-MRI radiomics), traditional (based on clinicopathologic and conventional MRI) and mixed (based on clinicopathologic, conventional MRI and DCE-MRI radiomics) models were constructed by logistic regression (LR) with a 3-fold cross-validation, all evaluated using receiver operating characteristic (ROC) curve analysis. A clinical radiomics nomogram was then built by incorporating the Radiomics score, significant clinicopathologic and conventional MRI features of mixed model.

**Results:**

The area under the curves (AUCs) of clinicopathologic, conventional MRI, DCE-MRI radiomics, traditional, combine, and mixed model were 0.76 (95% confidence interval [CI] 0.59–0.94), 0.77 (95%CI 0.59–0.95), 0.74 (95%CI 0.55–0.93), 0.87 (95%CI 0.73–1), 0.8 (95%CI 0.63–0.96), and 0.93 (95%CI 0.84–1) in the validation cohort, respectively. The clinical radiomics nomogram based on mixed model showed higher AUCs than both clinicopathologic and DCE-MRI radiomics models in training/test (all *P* < 0.05) set and showed the greatest overall net benefit for upstaging according to decision curve analysis (DCA).

**Conclusion:**

A nomogram constructed by combining clinicopathologic, conventional MRI features and DCE-MRI radiomics signatures may be useful in predicting DCISMI from DICS preoperatively.

**Supplementary Information:**

The online version contains supplementary material available at 10.1186/s12880-023-01092-5.

## Background

Breast cancer is one of the most common malignant tumors in women worldwide [1].With medical advances, the number of patients with ductal cancer in situ (DCIS) and DCIS with microinvasion (DCISMI) is increasing [2]. Malignant epithelial cell growth inside the mammary duct lumen but no penetration beyond the basement membrane is the histological hallmark of DCIS [3]. DCISMI is thought to be the transitional stage between DCIS and invasive ductal cancer (IDC) [4]. In this study, we used the AJCC’s definition of “microinvasion” as one foci (DCISMI-one) or more foci (DCISMI-more) of invasive carcinoma $\le$ 1 mm in diameter inside an area of DCIS [5]. Numerous studies indicate that DCISMI’s prognosis and natural history are quite similar to those of DCIS [6–10]. However, a large-scale clinical study [11] revealed that the prognosis of DCISMI is more like that of small invasive carcinoma than DCIS.

In comparison to mammography (MG) and ultrasonography (US), dynamic contrast enhanced MRI (DCE-MRI), a common procedure for breast MRI, offers the best sensitivity for identifying DCIS or DCIS coupled with invasive cancer [12]. It is critical to “identify MRI features that can be combined with clinical and biological characteristics to better stratify risk in patients with DCIS” [13]. Clinically, the majority of DCIS and DCISMI exhibit a clinically comparable morphological appearance. There have been a number of publications on characteristics helping to predict microinvasion and invasion, including clinical findings and findings on conventional imaging but with inconsistent results. However, there have been only a few publications that have compared MRI findings between pure DCIS and DCISMI cases [14, 15]. To the best of our knowledge, no previous report has researched the predictors of DCISMI using radiomics-based machine learning algorithms. A radiomics-based signature could provide a more thorough approach by combining both geographical and temporal data to define the tumor more fully. Thus, we speculate that radiomics characteristics obtained from DCE-MRI may represent cellular and molecular data and may be able to foretell upstaging in females with biopsy-proven DCIS.

In this study, we aimed to compare the performance of clinical-pathological characteristics, conventional breast MRI features, DCE-MRI radiomics signatures, and combined multiple features in predicting DCISMI and to construct a nomogram to better understand the risk factors.

## Materials and methods

### Patients

**Fig. 1:**
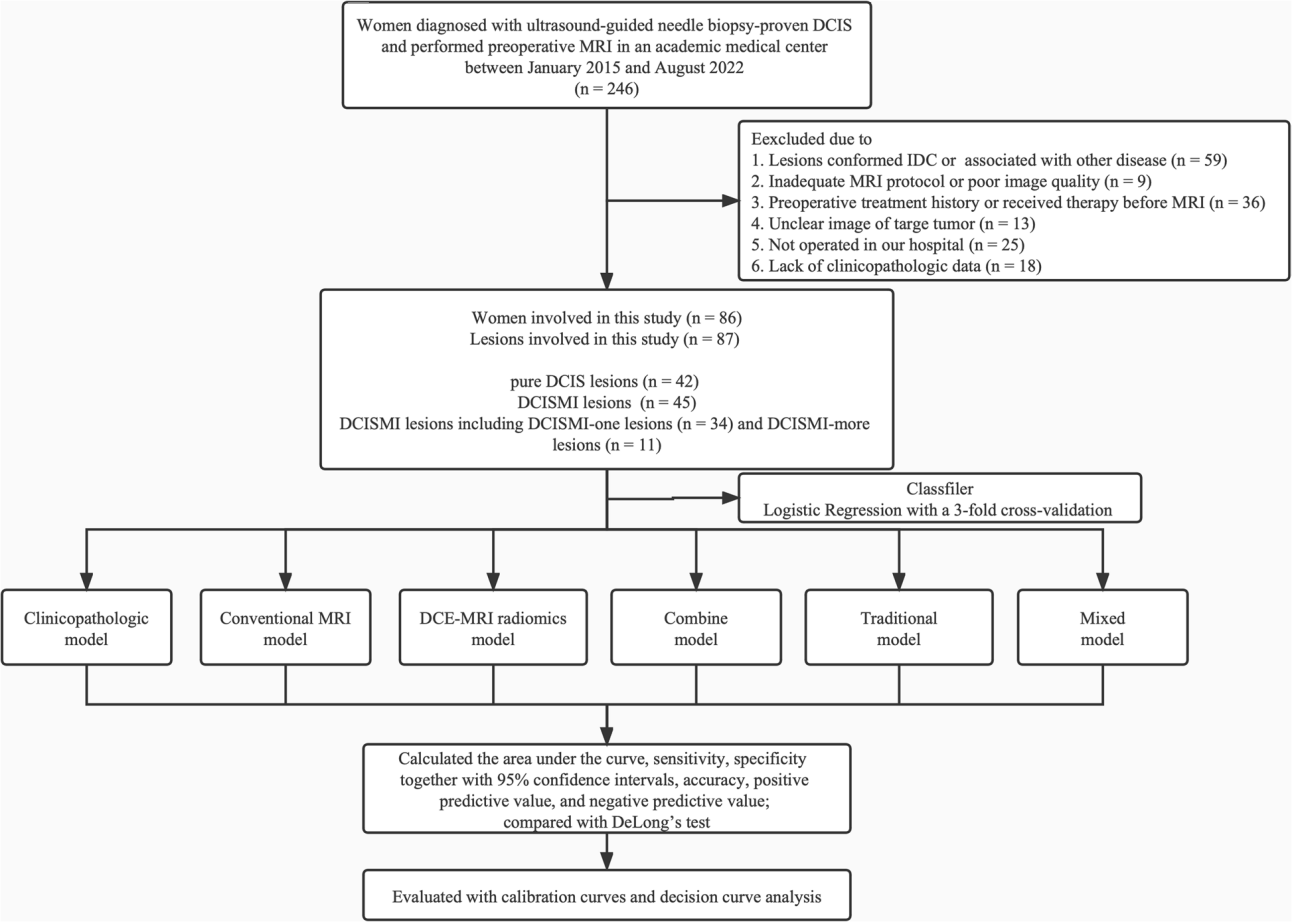
The flow diagram of the study. Abbreviations: DCIS, ductal carcinoma in situ; DCISMI, ductal carcinoma in situ with microinvasive; IDC, invasive ductal carcinoma; MRI, magnetic resonance imaging; DCE-MRI, dynamic contrast enhanced MRI; DCISMI-more, DCIS with multifocal of microinvasive carcinoma; DCISMI-one, DCIS with one focus of microinvasive carcinoma

The institutional ethics committee approved this retrospective study and granted a waiver of informed consent. We included 246 women who underwent preoperative breast MRI and US guided core needle biopsy (US-CNB) after MRI preoperatively with primary breast DCIS from January 2015 to August 2022. US-CNB was performed with a 14-guage automated biopsy gun (Stericut; TSK Laboratory, Tochigi, Japan) with five samples obtained from each lesion. The exclusion criteria were ① those who had IDC or were associated with another disease (*n* = 59); ② those who had an inadequate MRI protocol or poor image quality (*n* = 9); ③ those who had received therapy prior to the MRI (*n* = 36); ④ those whose tumors had unclear images (*n* = 13); ⑤ those who had not undergone surgery in our hospital (*n* = 25); ⑥ those who lacked clinicopathologic data (*n* = 18). This implies that the lesions were all US and MRI visible which would bias the series. Finally, the research cohort consisted of 86 individuals (mean age 44.30 ± 9.30 years) with 87 lesions (Fig. 1). One patient had DCIS that was verified by biopsy in both breasts.

### MRI Examinations

Two 3.0 T scanning systems (GE Discovery 750W and MAGNETOM Skyra, Siemens Healthcare), each with an eight-channel breast-specific coil, were used for all breast MRI scans. Women were in the prone position. The imaging protocol included a T2-weighted short tau inversion recovery turbo spin-echo pulse sequence (T2WI-FS) (repetition time [TR]/echo time [TE] = 4160/85 ms; matrix size = 512 × 512; field of view [FOV] = 350 × 350 mm^2^, section thickness = 5 mm for the GE 750W scanner; TR/TE = 3600/53 ms, matrix size = 320 × 320, FOV = 340 × 340 mm^2^, section thickness = 4 mm for the Simens Skyra scanner) and a DCE-MRI (TR/TE = 4.32/2.10; matrix size = 512 × 512; FOV = 350 × 350 mm^2^, section thickness = 0.7 mm for the GE 750W scanner; TR/TE = 4.49/1.68 ms, matrix size = 320 × 320, FOV = 340 × 340 mm^2^, section thickness = 1.2 mm for the Simens Skyra scanner). Gadolinium-DTPA (0.5 mmol/mL, Bayer) was power-injected at a dose of 0.1 mmol/kg body weight and a flow rate of 2.6 mL/s, followed by a 15 mL saline flush. The two scanners employed the same contrast agent and scanning mode. To balance the sample sizes on the two MRI protocols and to avoid the effect of excessive sample size bias on the results, we randomly selected the image data of above 86 patients with the same DICS and DCISMI component ratio.

### Data collection

Retrospective data retrieval from the hospital information system (HIS) database and the picture archiving and communication system (PACS) were performed on clinical-pathological and imaging data. Clinical-pathologic characteristics of the DCIS and DCISMI groups were shown in Table 1. The clinical-pathological including age, body mass intensity (BMI), DCIS grades of the biopsy specimen (low, intermediate, or high), the status of the estrogen receptor (ER), progesterone receptor (PR), and human epidermal growth factor receptor 2 (HER2), Ki-67 proliferation, p63 and axillary lymph node metastasis. HER2 positivity was defined as a protein overexpression score of 3 + determined by immunohistochemistry or the presence of gene amplification (positive in situ hybridization) [16]. Ki-67 scores $\ge$ 20% were considered high [9]. Conventional MRI features were included in Table 2, including lesion side, lesion number, lesion size on DCE-MRI scan ($\gg 2cm, <2cm$), and morphologic feature at T2WI-FS and DCE-MRI according to the Breast Imaging Reporting and Data System MRI lexicon. Intra tumoral high signal intensity (SI) [17] and peritumoral edema [18] were evaluated on T2WI-FS in this study. Reviewers were blinded to the clinicopathologic results.

**Table 1:** Clinicopathologic characteristics of DCIS in comparison with DCISMI in the study cohort

**Features**	**DCIS (*n* = 42)**	**DCISMI (*n* = 45)**	***P* value**
**MRI protocols**			0.36
GE	28(66.7%)	34(75.6%)	
SIEMENS	14(33.3%)	11(24.4%)	
**Age, years**	42.76 ± 8.742	45.71 ± 9.577	0.1382
**BMI**	23.25 ± 3.405	22.10 ± 2.548	0.0772
**ER status**			**0.0191**
Negative	12(28.57%)	24(53.33%)	
Positive	30(71.43%)	21(46.67%)	
**PR status**			**0.0074**
Negative	15(35.71%)	29(64.44%)	
Positive	27(64.29%)	16(35.56%)	
**HER2 status**			0.0878
Negative	15(35.71%)	8(17.78%)	
Positive	27(64.29%)	37(82.22%)	
**Ki-67 proliferation index (%)**			0.519
< 20	25(59.52%)	23(51.11%)	
≧20	17(40.48%)	22(48.89%)	
**p63**			**0.0004**
Negative	2(4.76%)	16(35.56%)	
Positive	40(95.24%)	29(64.44%)	
**Nuclear grade**			**0.0006**
Low	9(21.43%)	2(4.44%)	
Intermediate	16(38.1%)	7(15.56%)	
High	17(40.47%)	36(80%)	
**Axillary lymph node metastasis**			0.4948
Negative	42(100%)	43(95.56%)	
Positive	0	2(4.44%)	

Data values indicate the number of patients (with percentages in parentheses), or the mean ± standard deviation

*Abbreviations*: *DCIS* Ductal carcinoma in situ, *DCISMI* Ductal carcinoma in situ with microinvasive, *BMI* Body Mass Index, *ER* Estrogen receptor, *PR* Progesterone receptor, *HER2* Human epidermal growth factor receptor 2

**Table 2:** Conventional MRI characteristics in patients with DCIS in comparison with DCISMI

**Features**	**DCIS (*n* = 42)**	**DCISMI (*n* = 45)**	***P* value**
**Lesion side**			0.2821
Left	16(38.10%)	23(51.11%)	
Right	26(61.90%)	22(48.89%)	
**Types of mammary glands**			0.7581
a	1(2.38%)	1(2.22%)	
b	4(9.52%)	7(15.56%)	
c	33(78.58%)	31(68.89%)	
d	4(9.52%)	6(13.33%)	
**Lesion number**			0.4863
Single	39(92.86%)	39(86.67%)	
Two and Multiple	3(7.14%)	6(13.33%)	
**MR type of lesion**			0.6694
NME	19(45.42)	23(51.11%)	
Mass	23(54.76%)	22(48.89%)	
**Extent of DCIS (cm)**			0.813
< 2.0	13(30.95%)	12(26.67%)	
≧2.0	29(69.05%)	33(73.33%)	
**Intertumoral high SI on T2WI**			0.2302
No	2(4.76%)	0	
Yes	40(95.24%)	45(100.00%)	
**Peritumoral edema on T2WI**			**0.0022**
No	20(47.62%)	7(15.56%)	
Yes	22(52.38%)	38(84.44%)	
**Initial enhancement**			0.1883
Slow	38(90.48%)	44(97.78%)	
Medium	3(7.14%)	0	
Fast	1(2.38%)	1(2.22%)	
**Enhancement peak (mean ± SD)**	1537 ± 206.1	1574 ± 231.9	0.4417
**Delayed enhancement**			0.2647
Persistent	9(21.43%)	11(24.44%)	
Plateau	30(71.43%)	26(57.78%)	
Washout	3(7.14%)	8(17.78%)	
^a^**Heterogeneous enhancement pattern**			** < 0.0001**
No	24(57.14%)	3(6.67%)	
Yes	18(42.86%)	42(93.33%)	
**Necrosis within lasion**			0.5756
No	36(85.71%)	36(80.00%)	
Yes	6(14.29%)	9(20.00%)	
**BI-RADS**			0.9046
2	1(2.38%)	2(4.44%)	
3	4(9.52%)	4(8.89%)	
4	32(76.19%)	32(71.11%)	
5	5(11.91%)	7(15.56%)	
**NAC invasion on MRI**			0.4948
No	42(100.00%)	43(95.56%)	
Yes	0	2(4.44%)	
**Contralateral occult lesion on MRI**			0.0631
No	33(78.57%)	42(93.33%)	
Yes	9(21.43%)	3(6.67%)	

Data indicate the number of lesions (with percentages in parentheses) or the mean ± standard deviation

*Abbreviations*: *DCIS* Ductal carcinoma in situ, *DCISMI* Ductal carcinoma in situ with microinvasive, *SI* Signal intensity, *T2WI* T2-weighted image, *NME* Non-mass enhancement, *NAC* Nipple-areolar complex

^a^Heterogeneous enhancement pattern includes heterogeneous, clumped, and clustered ring pattern

Medical picture registration was done using ITK-SNAP programmed software (version 3.4.0; http://www.itksnap.org). In the phase of the contrast-enhanced acquisition, which clearly displayed the lesion, two radiologists with five years of experience delineated the tumor region of interest (ROI) along the edge of the lesion in each layer, and then duplicated the ROIs for the remaining four acquisitions. The third radiologist with 10 years of experience reexamined and validated the final boundary after the discussion when there was a significant discrepancy between the two radiologists. This method produced accurate feature extraction and accurate tumor outlines. We chose the sets of all the lesions completed by the two radiologists (5 years of work experience) to assess the repeatability of radiomics features. The interobserver reproducibility of feature extraction was assessed by the intra-class correlation coefficient (ICC). ICC ≥ 0.75 indicated high consistency and selected for further investigation.

### Establishment models

Firstly, selectkbest (*P* $<$ 0.05) was used to select the optimal predictive features to establish the clinicopathologic and conventional MRI models by the logistic regression (LR) classifier based on clinicopathologic characteristics and conventional MRI features, respectively.

**Fig. 2:**
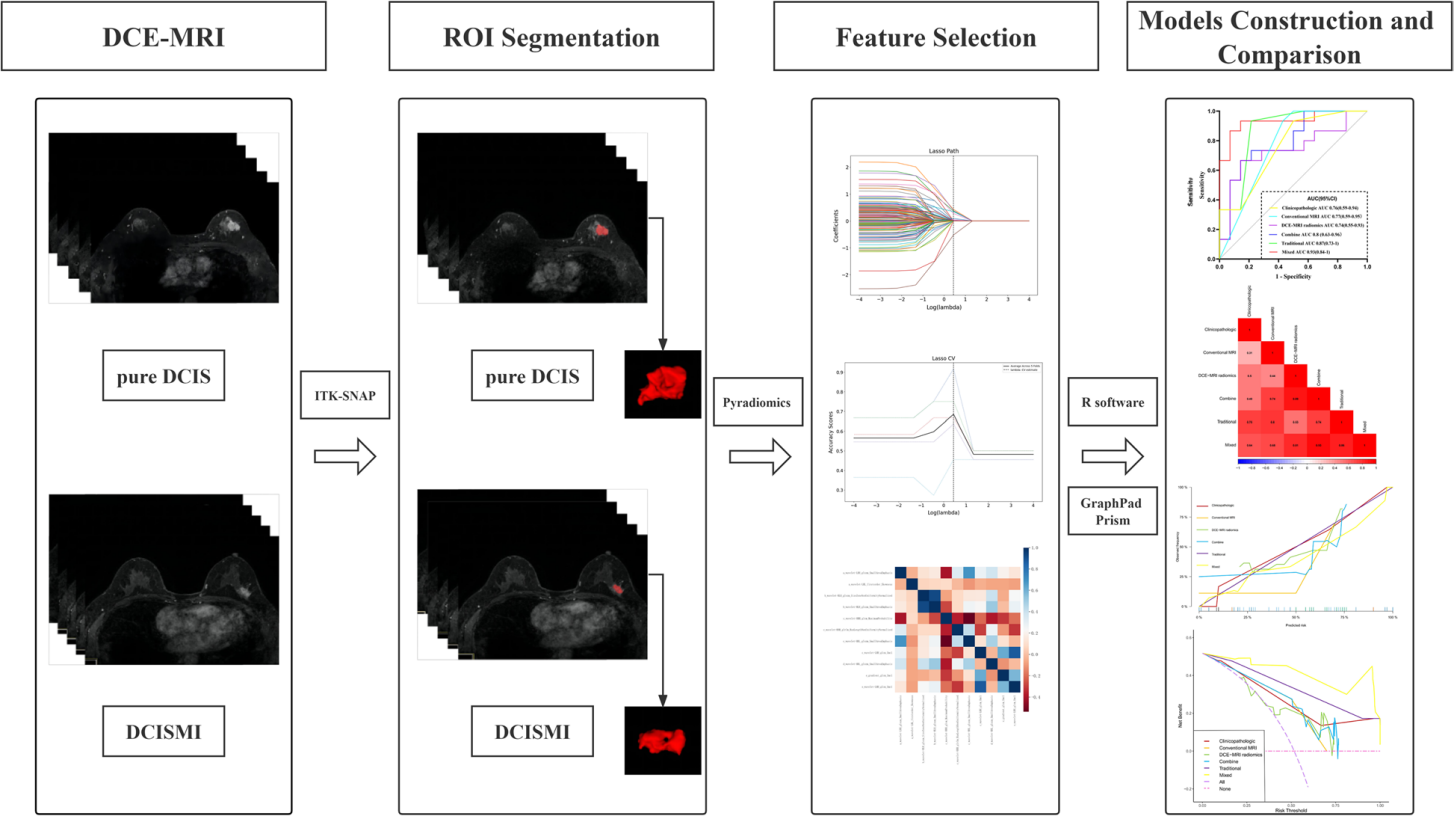
The radiomics flow chart of the study. Abbreviations: DCIS, ductal carcinoma in situ; DCISMI, ductal carcinoma in situ with microinvasive; MRI, magnetic resonance imaging; DCE-MRI, dynamic contrast enhanced MRI

PyRadiomics (version 3.0.1; http://github.com/Radiomics/pyradiomics) was used to extract radiomics features of DCE-MRI. As the images were derived from two MRI scanners with different parameters, normalization was performed before features could be extracted from the ROIs of the DCE-MRI images. To facilitate consistent feature extraction, the image data were normalized and preprocessed in the following steps: spatial resampling to 1 × 1 × 1 mm^3^ and intensity discretization to a fixed bin width of 25. Radiomics features can be calculated on the pre-processed images using the wavelet and Laplacian of Gaussian (LoG) flters with varying sigma (= 1.0, 2.0, 3.0, 4.0, 5.0). To reduce overfitting or selection bias, Select Percentile (*P* $<0.05$) and least absolute shrinkage selection operator (LASSO) were used to further select the optimal predictive features to establish the DCE-MRI radiomics model. Shape-based, first-order statistical, gray-level cooccurrence matrix (GLCM), gray-level region matrix (GLSZM), gray-level run-length matrix (GLRLM), and gray-level dependence matrix (GLDM) were extracted from original and filtered images for a total of 7046 features. The workflow is presented in Fig. 2. The clinicopathologic, conventional MRI, DCE-MRI radiomics, combine (including conventional MRI and DCE-MRI radiomics), traditional (including clinicopathologic and conventional MRI) and mixed (including clinicopathologic, conventional MRI and DCE-MRI radiomics) models were constructed by the LR classifier with a threefold cross-validation, to ensure that it was not affected by insufficient sample size. Meanwhile, we also analyzed whether the DCE-MRI radiomics model could distinguish DCISMI-more from DCISMI-one.

### Statistical analysis

Clinicopathologic and conventional MRI features were compared between the DCIS and DCISMI groups using the Chi-square or Fisher’s exact test for categorical variables and the analysis of variance (ANOVA) or Kruskal–Wallis test for continuous variables.

All statistical analyses were performed with SPSS software version 20.0 (SPSS, Chicago, IL, USA). A two-sided *P* $<$ 0.05 was considered statistically significant. To evaluate the predictive ability of different models, the area under the curve (AUC) of the receiver operating curve (ROC), sensitivity (SEN), specificity (SPE) with 95% confidence interval (CI), accuracy (ACC), positive predictive value (PPV), and negative predictive value (NPV) were calculated together by using a given cutoff of the predicted probability of DCISMI and distinguished DCISMI-more from DCISMI-one by the DCE-MRI radiomics model as well. AUCs between different models were compared using DeLong’s test. Calibration curves were employed to assess the predictive performance of each model. To evaluate each model’s clinical applicability, decision curve analysis (DCA) was used.

## Results

### Clinicopathologic and conventional MRI features assessment

Among the 87 biopsy-confirmed DCIS lesions, 42 (48.28%) were found to be pure DCIS in the final surgical pathology, and 45 (51.72%) were upgraded to DCISMI. The mean age at diagnosis and BMI did not significantly differ between the two groups. Histologically, higher nuclear grade (*P* = 0.0006), negative p63 (*P* = 0.0004), negative ER (*P* = 0.0191), and negative PR (*P* = 0.0074) were more significantly common in DCISMI group. The DCISMI group tended to show higher positive HER2 than the DCIS group; however, this difference did not reach statistical significance (*P* = 0.0878). The clinicopathologic features of these samples are summarized in Table 1.

Table 2 presents the conventional MRI features of the two groups. The DCISMI group tended to show more peritumoral edema (*P* = 0.0022) and heterogeneous enhancement (*P* < 0.0001) frequent than the DCIS group. There were no significant differences between groups aside from above the two features of conventional MRI.

**Fig. 3:**
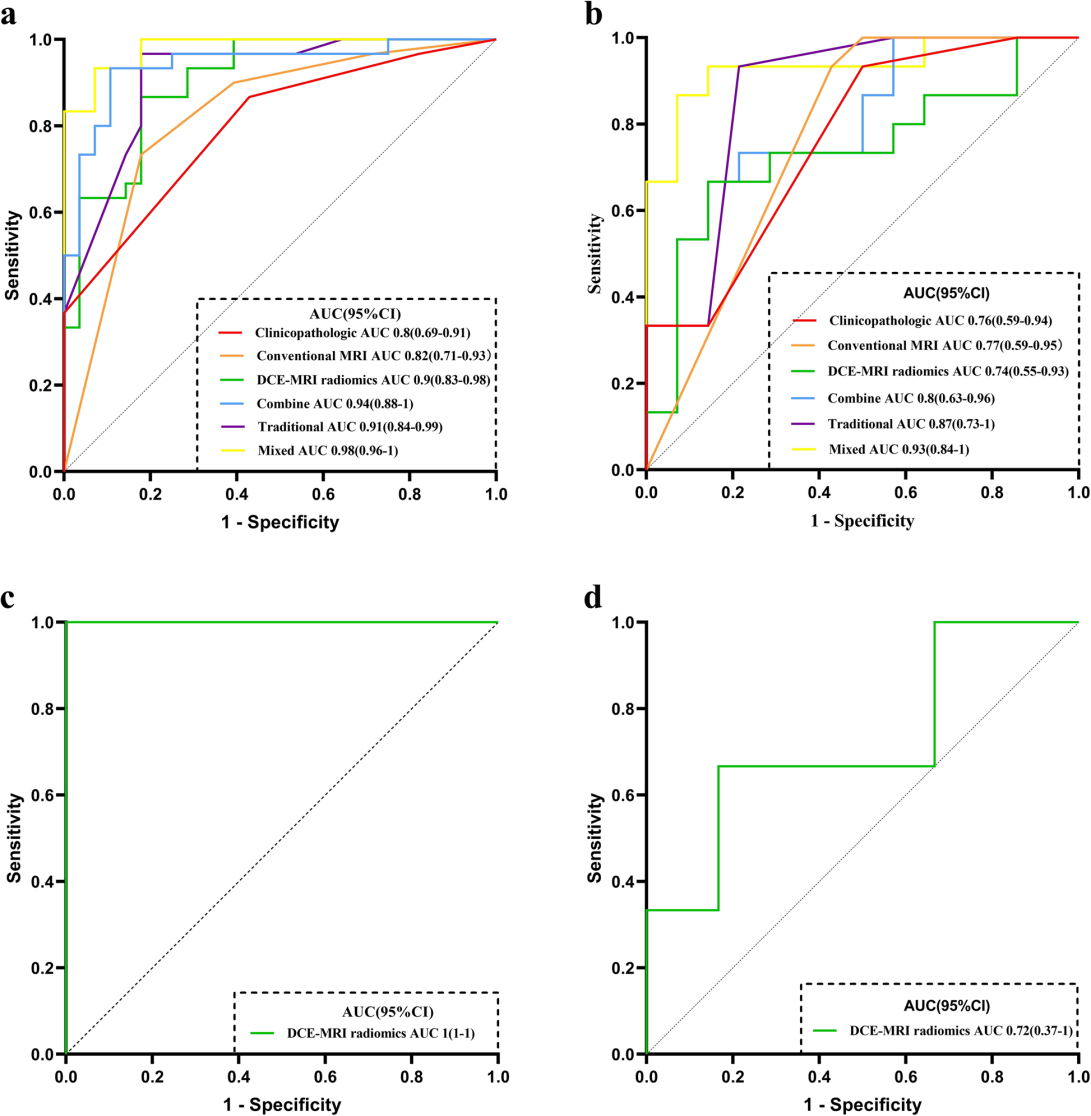
The receiver operating characteristic curves (ROC) of the six models in the upstaging of DICS. **a** The ROC curves of clinicopathologic, conventional MRI, DCE-MRI radiomics, combine, traditional, and mixed models based on LR algorithm in the training cohort. **b** The ROC curves of clinicopathologic, conventional MRI, DCE-MRI radiomics, combine, traditional, and mixed models based on LR algorithm in the test cohort. **c** The ROC curve of the DCE-MRI radiomics model in distinguishing DCISMI-more from DCISMI-one in the training cohort. **d** The ROC curve of the DCE-MRI radiomics model in distinguishing DCISMI-more from DCISMI-one in the test cohort. Abbreviations: ROC, receiver operating characteristic curves; DCIS, ductal carcinoma in situ; MRI, magnetic resonance imaging; DCE-MRI, dynamic contrast enhanced MRI; LR, logistic regression; CI, confidence interval; AUC, area under the curve; DCISMI-more, DCIS with multifocal of microinvasive carcinoma; DCISMI-one, DCIS with one focus of microinvasive carcinoma

Among all the clinicopathologic and conventional MRI features, variables were selected by the selectkbest to establish in subsequent machine learning by the LR classifier (Table 3). The results revealed that higher nuclear grade (odds ratio [OR] 3.208, 95% confidence interval [CI]1.122–9.176) was the only independent factor associated with histologic upgrade in the clinicopathologic model, and peritumoral edema (OR 4.098, 95%CI 1.061–15.832) and heterogeneous enhancement (OR 14.112, 95%CI 3.231–61.631) remained significant independent factors in the conventional MRI model. However, the heterogeneous enhancement pattern remained the only significant independent factor in the traditional model (OR 28.243, 95%CI 3.053–261.251). The primary features extracted in each model and LR results for each model in predicting DCISMI with OR were shown in the Appendix Table 1. The prediction performance of clinicopathologic, conventional MRI and traditional models were shown in Table 4 and Fig. 3a, b with AUCs of 0.8 (95%CI 0.69–0.91)/0.76 (95%CI 0.59–0.94), 0.82 (95%CI 0.71–0.93)/0.77 (95%CI 0.59–0.95), and 0.91 (95%CI 0.84–0.99)/0.87 (95%CI 0.73–1) in training/test set, respectively.

**Table 3:** The features selection methods and their corresponding parameters in the models

**Models**	**Features**	**SelectKBest** ***P* value**	**SelectKBest Score**	**Select Percentile** ***P* value**	**Select Percentile Score**	**Lasso coefficient**
**Clinicopathologic**	p63	0.000216545	15.65154265	NA	NA	NA
	nuclear grade	0.023386159	5.432974831	NA	NA	NA
**Conventional MRI**	heterogeneous enhancement pattern	0.0000164	22.2414581	NA	NA	NA
	peritumoral edema on T2WI	0.015883232	6.185856224	NA	NA	NA
**DCE-MRI radiomics**	a_wavelet-LHH_glszm_SmallAreaEmphasis	NA	NA	0.010987487	6.920084579	0.001458018
	a_wavelet-LHL_firstorder_Skewness	NA	NA	0.017251576	6.023456627	-0.522407251
	b_wavelet-HLH_glszm_SizeZoneNonUniformityNormalized	NA	NA	0.001811074	10.73097267	0.441698208
	b_wavelet-HLH_glszm_SmallAreaEmphasis	NA	NA	0.000770171	12.65733984	0.350393762
	c_wavelet-HHH_glcm_MaximumProbability	NA	NA	0.005073203	8.510581486	-0.006136234
	c_wavelet-HHH_glrlm_RunLengthNonUniformityNormalized	NA	NA	0.038189923	4.507011169	0.275763724
	c_wavelet-HHL_glszm_SmallAreaEmphasis	NA	NA	0.002185117	10.31798937	0.346955315
	c_wavelet-LHH_glcm_Imc1	NA	NA	0.033946605	4.726340662	0.02556862
	d_wavelet-HHL_glszm_SmallAreaEmphasis	NA	NA	0.006421524	8.018453923	0.04114306
	e_gradient_glcm_Imc1	NA	NA	0.028745645	5.039358324	0.379574092
	e_wavelet-LHH_glcm_Imc1	NA	NA	0.031984038	4.837987471	0.089713532

*Abbreviations*: *DCE-MRI* Dynamic contrast enhanced MRI, *NA* Not available

a, b, c, d and e represent phase 1, 2, 3, 4 and 5 of dynamic enhancement, respectively

**Table 4:** Predictive performances of the six models and DCE-MRI radiomics model in distinguishing DCISMI-more from DICSMI-one

**Feature number**	**Models**	**Method**	**AUC(95% CI)**	**SEN (95% CI%)**	**SPE (95% CI%)**	**ACC**	**PPV**	**NPV**
2	Clinicopathologic	test set	0.76(0.59–0.94)	0.93(0.7–0.99)	0.5(0.27–0.73)	0.72	0.67	0.88
		training set	0.8(0.69–0.91)	0.87(0.7–0.95)	0.57(0.39–0.73)	0.72	0.68	0.8
2	Conventional MRI	test set	0.77(0.59–0.95)	0.93(0.7–0.99)	0.57(0.33–0.79)	0.76	0.7	0.89
		training set	0.82(0.71–0.93)	0.73(0.56–0.86)	0.82(0.64–0.92)	0.78	0.81	0.74
11	DCE-MRI radiomics	test set	0.74(0.55–0.93)	0.73(0.48–0.89)	0.71(0.45–0.88)	0.72	0.73	0.71
		training set	0.9(0.83–0.98)	0.87(0.7–0.95)	0.82(0.64–0.92)	0.84	0.84	0.85
13	^a^Combine	test set	0.8 (0.63–0.96)	0.73(0.48–0.89)	0.64(0.3–0.84)	0.69	0.69	0.69
		training set	0.94(0.88–1)	0.93(0.79–0.99)	0.89(0.73–0.96)	0.91	0.9	0.93
4	^b^Traditional	test set	0.87(0.73–1)	1(0.8–1)	0.36(0.16–0.61)	0.69	0.63	1
		training set	0.91(0.84–0.99)	0.97(0.83–1)	0.82(0.64–0.92)	0.9	0.85	0.96
15	^c^Mixed	test set	0.93(0.84–1)	0.93(0.7–0.99)	0.5(0.27–0.73)	0.72	0.67	0.88
		training set	0.98(0.96–1)	0.93(0.79–0.99)	0.93(0.77–0.99)	0.93	0.93	0.93
16	DCE-MRI radiomics predicting DCISMI-more	test set	0.72(0.37–1)	0.67(0.12–0.98)	0.83(0.55–0.97)	0.8	0.5	0.91
		training set	1(1–1)	1(0.65–1)	1(0.86–1)	1	1	1

*Abbreviations*: *DCISMI* Ductal carcinoma in situ with microinvasive, *DCISMI-more* DCIS with multifocal of microinvasive carcinoma, *DCISMI-one* DCIS with one focus of microinvasive carcinoma, *AUC* Area under the curve, *SEN* Sensitivity, *SPE* Specificity, *ACC* accuracy, *PPV* Positive predictive value, *NPV* Negative predictive value, *CI* Confidence interval, *DCE-MRI* Dynamic-contrast enhanced MRI

^a^Combine model was constructed based on conventional MRI and DCE-MRI radiomics features

^b^Traditional model was constructed based on clinicopathologic and conventional MRI features

^c^Mixed model was constructed based on clinicopathologic, conventional MRI and DCE-MRI radiomics features

### DCE-MRI radiomics assessment

Eleven radiomics features were independent predictors of upstaging after feature selection (Table 3). The results revealed that the radiomics score was one of the significant independent factors associated with histologic upgrade in the DCE-MRI radiomics model (OR 639.215, 95%CI 32.954–14582.214), combine model (OR 475.328, 95%CI 17.018–13276.428), and mixed model (OR 1106.221, 95%CI 10.104–121118.327), respectively. The other significant independent factor in the mixed model was heterogeneous enhancement (OR 33.327, 95%CI 2.317–479.287). The primary features extracted in each model and LR results for each model in predicting DCISMI with OR were shown in the Appendix Table 1. The prediction performance of the DCE-MRI radiomics, combine, and mixed models were shown in Table 4 and Fig. 3a, b with AUC values of 0.9 (95%CI 0.83–0.98)/0.74 (95%CI 0.55–0.93), 0.94 (95%CI 0.88–1)/0.8 (95%CI 0.63–0.96), and 0.98 (95%CI 0.96–1)/0.93 (95%CI 0.84–1) in training/test sets, respectively. The AUC of the DCE-MRI radiomics model in distinguishing between DCISMI-one (*n* = 34) and DCISMI-more (*n* = 11) was 1 (95%CI 1–1)/0.72 (95%CI 0.37–1) in training/test set (Table 4 and Fig. 3c, d).

### Model comparisons and establishment of a preoperative nomogram

**Fig. 4:**
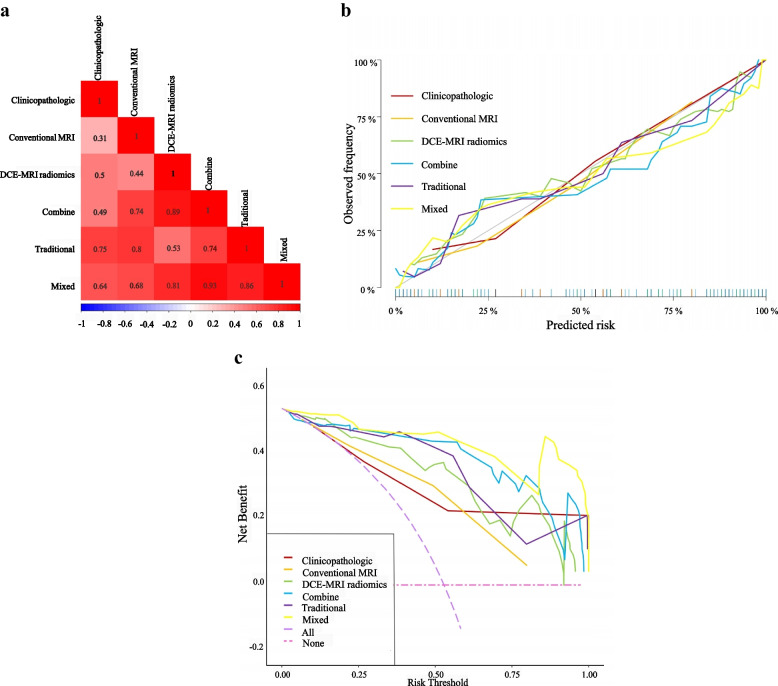
The heatmap, calibration curves and DCA of the six models. **a** Heatmap comparison of the clinicopathologic, conventional MRI, DCE-MRI radiomics, combine, traditional, and mixed models. **b** Calibration curves for the clinicopathologic, conventional MRI, DCE-MRI radiomics, combine, traditional, and mixed models based on the LR algorithm. It is the curve with the model-predicted probability of DCISMI as the X-axis and the actual rate acquired by the bootstrapping method as the Y-axis. The degree of agreement between the depicted calibration curve and the 45° straight line reflects the predictive performance of each model. **c** The DCA for the clinicopathologic, conventional MRI, DCE-MRI radiomics, combine, traditional, and mixed models based on the LR algorithm. The Y-axis represents the net benefit. DCA showed that in six models within reasonable threshold probabilities, the mixed model showed the greatest overall net benefit for upstage and the second was the combine model. The DCE-MRI radiomics model, which showed all but the same net benefit as the traditional model, showed better than the conventional MRI model. The combine model added more net benefit than the traditional model at the range of 0.4 ~ 1.0. The clinicopathologic model added more net benefit than the conventional MRI model and DCE-MRI radiomics model from 0.65 to 1.0 and from 0.7 to 1.0, respectively. Abbreviations: DCA, decision curve analysis; MRI, magnetic resonance imaging; DCE-MRI: dynamic contrast enhanced MRI; LR, logistic regression

DeLong’s test showed that the traditional model achieved higher AUCs than the clinicopathologic model in both the training and test sets (all *P* < 0.05). The mixed model showed better AUCs than both the clinicopathologic and DCE-MRI radiomics models in both the training and test sets as well (all *P* < 0.05). A comparison of the six models is shown as a heat map in Fig. 4a.

**Fig. 5:**
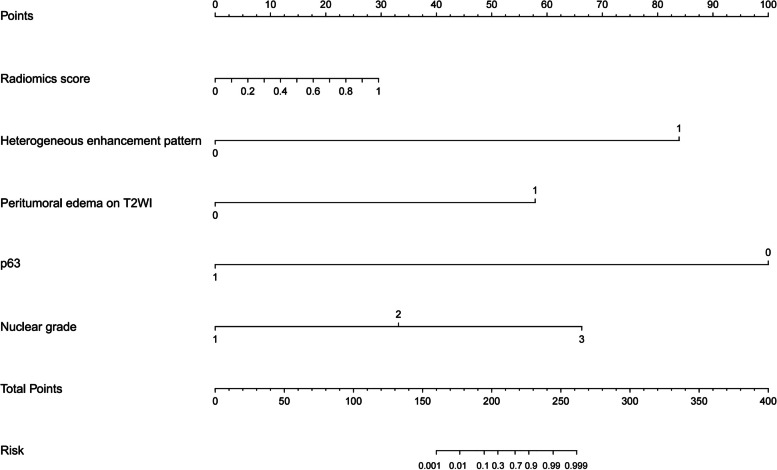
The nomogram combining nuclear grade, p63, peritumoral edema, heterogeneous enhancement pattern and radiomics scores. The clinical radiomics nomogram was developed for the prediction of DCISMI with nuclear grade, p63, peritumoral edema on T2WI, heterogeneous enhancement pattern, and radiomics scores. Abbreviations: T2WI, T2-weighted image; DCISMI, ductal carcinoma in situ with microinvasive

The calibration curves of the models are shown in Fig. 4b, which shows good calibration. DCA (Fig. 4c) illustrated that the mixed model showed the greatest overall net benefit for upstage and the second was the combine model within reasonable threshold probabilities. The DCE-MRI radiomics model, which showed all but the same net benefit as the traditional model, showed better than the conventional MRI model. To provide a visualized outcome measure, a preoperative nomogram figure was plotted based on training cohort by combining the p63, nuclear grade, peritumoral edema on T2WI, heterogeneous enhancement pattern and Radiomics score in Fig. 5.

## Discussion

Our preliminary analysis showed that the preoperative clinicopathologic, conventional breast MRI and DCE-MRI radiomics features could predict the preoperative histological upstage of DCIS and that DCE-MRI radiomics signatures may be different for DCISMI-more from DCISMI-one. The mixed model showed excellent predictive performance. The DCE-MRI radiomics model, which could be replaced by the traditional model, showed better than the conventional MRI model. A nomogram constructed by combining clinicopathologic, conventional MRI features and DCE-MRI radiomics signatures may be useful in predicting DCISMI from DICS, preoperatively.

DCISMI represents an intermediate state between DCIS and IDC [19]. Although DCIS, DCISMI, and T1a IDC (invasive tumor size $>$ 0.1 cm but $\le$ 0.5 cm in greatest dimension was classified as T1a) all have generally excellent prognosis, some studies revealed that DCISMI more closely resembles small invasive carcinoma than pure DCIS, and many practitioners are treating it as such [11, 20]. In comparison to women with pure DCIS or DCISMI-one, DCISMI-more is linked to a higher probability of invasive local recurrence in women with DCIS treated with breast conserving surgery [21]. The rate of axillary lymph node metastasis is approximately 0% to 20% in DCISMI [19]. According to approved treatment guidelines, sentinel lymph node biopsy was used in conjunction with mastectomy because of these relatively low but clinically significant rates [22]. A change in diagnosis from pure DCIS before surgery to DCISMI after surgery creates a great deal of patient anxiety and possibly a second surgery [23]. Therefore, an accurate prediction of the histological DCISMI and even DICSMI-more could help with preoperative risk stratification and the best choice of patients who could benefit from more extensive surgery while avoiding overtreatment of patients at low risk.

In this study, higher nuclear grade as the independent factor associated with histologic upgrade in the clinicopathologic model and both the peritumoral edema and heterogeneous enhancement as significant independent factors in the conventional MRI model, which were partly consistent with those of previous studies for predicting invasive breast cancer [15, 24–27]. Several prior studies have described the MRI features [14, 15, 28–32] that can help predict the invasive component of a biopsy-proven DCIS. However, there have been few reports that have compared MRI findings between pure DCIS and DCISMI, rarely by the features using machine learning algorithms based on the clinicopathologic, conventional breast MRI, and DCE-MRI radiomics signatures. In this study, the mixed model established by the LR classifier showed that heterogeneous enhancement pattern and Radiomics score were independent predictors of upstage. DCISMI lesions showed more heterogeneous enhancement, which is partly consistent with previous studies [15, 28, 33]. The main applications of breast radiomics investigations include the molecular categorization, lymph node metastases, and molecular markers of IDC and DCIS prediction [34]. There is only one report based on US radiomics to predict the molecular biomarkers of DICS [35]. Radiomics is a precision medical method for non-invasive diagnosis, evaluation of efficacy, and biological behavior [36]. Contrary to our prior hypothesis, it was found that the DCE-MRI radiomics model’s AUC (AUC = 0.74) was the lowest one below 0.8. This may be because the MRI radiomics mostly depend on a set of MRI sequences [35]. In our study, only DCE-MRI radiomics features were included rather than diffusion weighted imaging (DWI) or apparent diffusion coefficient (ADC) radiomics features. DWI may provide more accurate tumor microenvironment monitoring [37], which has been widely explored to differentiate benign from malignant breast lesions [38, 39], and showed its diagnostic ability in DCISMI [33] and predicting upstaging of invasive components in biopsy-proven DCIS [29, 40]. Because some lesions were not visualized on DWI, DWI sequences were not included in this study. In our investigation, the DCE-MRI radiomics model was able to distinguish between DCISMI-more and DCISMI-one with an AUC of 0.72 and an accuracy of 0.8 using a very small sample size of 11 DCISMI-more lesions and 34 DCISMI-one lesions. All patients underwent sentinel lymph node surgery in this study. Patients with a final diagnosis of pure DCIS and DCISMI-one have no axillary lymph node metastasis in our institution, while there were 2 cases (4.4%) of DCISMI-more patients with axillary lymph node metastasis. DCE-MRI radiomics potential prediction in DICSMI-more and the relationship between DCISMI-more and axillary lymph node metastasis should be verified by future prospective multicenter studies with larger samples.

Our research revealed that, with a histologic upgrade rate somewhat higher than that of other studies with 8.8%-51.5% of DCIS patients upstaged to invasive disease [15, 41], 51.72% of lesions having a preoperative diagnosis of DCIS were histopathologically upgraded to DCISMI. There might be a few reasons. Firstly, the study’s sample size was small. Secondly, patients with DCIS histopathologically upgraded to IDC were excluded from our analysis according to the study’s objectives. Thirdly, the lesions were all US and MRI visible which would bias the series toward a higher rate of microinvasive cancer. To date, few features have consistently surfaced as strong predictors of upstaging on excision.

We discovered that the mixed model performed exceptionally well for preoperatively predicting the histological upstage of DCISMI. In our investigation, they were combined into a unique clinicopathologic + conventional MRI + radiomics nomogram, which demonstrated sufficient prediction performance. The clinicopathologic prediction model reflected the role of clinical and pathological baseline information in upstage prediction. While the radiomics model based on DCE-MRI included quantification of pictures, the conventional MRI model based on T2WI-FS and DCE-MRI involved qualitative assessment of images. The mixed model nomogram has the potential to increase diagnostic effectiveness and net benefit over the whole spectrum of threshold probabilities in DCA, in addition to displaying and customizing the likelihood of DCISMI for each patient.

This study had several limitations that should be noted. First, this was a retrospective study with a relatively small sample size at a single center. Second, two different breast MRI protocols (3.0 T Siemens and GE) were used at our hospital during the study period. At the same time, this may reflect the stability of the models in our study. Third, the DWI results were not examined, which would have revealed more data. Fourth, it was difficult to draw the margin of some DCIS lesions with non-mass enhancement. Finally, we did not perform an external validation test using an independent data set, although a 3-fold cross-validation was used. To verify our nomogram, additional external validation utilizing several independent data sets would be required.

## Conclusion

Our preoperative nomogram model specifically for DCISMI patients with clinicopathologic, conventional MRI, and DCE-MRI radiomics signatures enabled a more accurate prediction of upstaging in women with biopsy-proven DCIS. This could help to select women who were indicated for sentinel lymph node biopsy at initial surgery, thus avoiding unnecessary axillary surgery and preventing delayed secondary surgery. Although validation requires a larger sample size, DCE-MRI radiomics may discriminate between DCISMI-more and DCISMI-one.

### Supplementary Information

**Additional file 1: Appendix Table 1.** The primary features extracted in each model in predicting DCISMI.

## Data Availability

The datasets used and/or analyzed during the current study are available from the corresponding author on reasonable request.

## Abbreviations

DCIS: Ductal carcinoma in situ
DCISMI: Ductal carcinoma in situ with microinvasive
DCISMI-more: DCIS with multifocal of microinvasive carcinoma
DCISMI-one: DCIS with one focus of microinvasive carcinoma
IDC: Invasive ductal carcinoma
T2WI: T2-weighted image
T2WI-FS: T2-weighted fat-suppressed turbo spin echo
MG: Mammography
US: Ultrasound
MRI: Magnetic resonance imaging
DCE-MRI: Dynamic contrast enhanced MRI
DWI: Diffusion-weighted imaging
ADC: Apparent diffusion coefficient
ANOVA: Analysis of variance
LASSO: Least absolute shrinkage selection operator
LR: Logistic regression
NME: Non-mass enhancement
SI: Signal intensity
ER: Estrogen receptor
PR: Progesterone receptor
HER2: Human epidermal growth factor receptor 2
ROI: Region of interest
GLCM: Gray level co-occurrence matrix
GLRLM: Gray level run-length matrix
GLSZM: Gray-level size-zone matrix
GLDM: Gray-level dependence matrix
NGTDM: Neighborhood gray tone difference matrix
ROC: Receiver operating characteristic
AUC: Area under the curve
DCA: Decision curve analysis
CI: Confidence interval
ACC: Accuracy
PPV: Positive predictive value
NPV: Negative predictive value
OR: Odds ratio
BMI: Body Mass Index
NAC: Nipple-areolar complex
TR: Repetition time
TE: Echo time
FOV: Field of view
HIS: Hospital information system
PACS: Picture archiving and communication system
ICC: Intra-class correlation coefficient
