# Supplementary material for: A clinical radiomics nomogram preoperatively to predict ductal carcinoma in situ with microinvasion in women with biopsy-confirmed ductal carcinoma in situ: a preliminary study
Source: BMC Med Imaging. 2023 Sep 7;23:118. doi: 10.1186/s12880-023-01092-5 (PMC10483851; doi:10.1186/s12880-023-01092-5)
Supplement: Supplementary file 1 — Additional file 1: Appendix Table 1. The primary features extracted in each model in predicting DCISMI. [file 12880_2023_1092_MOESM1_ESM.docx]

**Appendix Table 1** The primary features extracted in each model in predicting DCISMI.

| **Features** | | **Clinicopathologic** | **Conventional MRI** | **DCE-MRI radiomics** | **Combine** | **Traditional** | **Mixed** |
| --- | --- | --- | --- | --- | --- | --- | --- |
|  |  | **OR (95%CI)** | **OR (95%CI)** | **OR (95%CI)** | **OR (95%CI)** | **OR (95%CI)** | **OR (95%CI)** |
| **Clinicopathologic** | p63 | 0 | NA | NA | NA | 0 | 0 |
|  | Nuclear grade | 3.208(1.122-9.176) | NA | NA | NA | 2.526(0.787-8.106) | 3.007(0.409-22.124) |
| **Conventional MRI** | Peritumoral edema on T2WI | NA | 4.098(1.061-15.832) | NA | 2.851(0.496-16.38) | 3.143(0.594-16.627) | 4.498(0.474-42.715) |
|  | Heterogeneous enhancement pattern | NA | 14.112(3.231-61.631) | NA | 11.487(1.766-74.722) | 28.243(3.053-261.251) | 33.327(2.317-479.287) |
| **DCE-MRI radiomics** | Radiomics score | NA | NA | 639.215(32.954-14582.214) | 475.328(17.018-13276.428) | NA | 1106.221(10.104-121118.327) |
|  | a_wavelet-LHH_glszm_SmallAreaEmphasis | NA | NA | 0.918(0.235-3.585) | 0.675(0.071-6.418) | NA | 0.975(0.088-10.751) |
|  | a_wavelet-LHL_firstorder_Skewness | NA | NA | 0.309(0.1-0.951) | 0.832(0.196-3.533) | NA | 0.612(0.107-3.488) |
|  | b_wavelet-HLH_glszm_SizeZoneNonUniformityNormalized | NA | NA | 5.361(0.189-151.966) | 14.454(0.14-1491.859) | NA | 43.232(0.028-67856.137) |
|  | b_wavelet-HLH_glszm_SmallAreaEmphasis | NA | NA | 1.075(0.04-29.016) | 0.338(0.004-31.263) | NA | 0.151(0-90.781) |
|  | c_wavelet-HHH_glcm_MaximumProbability | NA | NA | 1.264(0.341-4.679) | 1.926(0.287-12.927) | NA | 2.748(0.344-21.954) |
|  | c_wavelet-HHH_glrlm_RunLengthNonUniformityNormalized | NA | NA | 2.389(0.653-8.736) | 2.115(0.407-10.981) | NA | 1.617(0.304-8.613) |
|  | c_wavelet-HHL_glszm_SmallAreaEmphasis | NA | NA | 1.871(0.385-9.094) | 2.455(0.372-16.208) | NA | 3.011(0.444-20.409) |
|  | c_wavelet-LHH_glcm_Imc1 | NA | NA | 1.201(0.205-7.029) | 1.024(0.113-9.275) | NA | 0.632(0.056-7.126) |
|  | d_wavelet-HHL_glszm_SmallAreaEmphasis | NA | NA | 1.198(0.307-4.673) | 4.429(0.495-39.61) | NA | 5.144(0.488-54.213) |
|  | e_gradient_glcm_Imc1 | NA | NA | 3.102(0.542-17.765) | 2.248(0.316-15.975) | NA | 1.655(0.173-15.825) |
|  | e_wavelet-LHH_glcm_Imc1 | NA | NA | 1.587(0.269-9.371) | 2.514(0.234-26.992) | NA | 6.29(0.287-138.009) |

Abbreviations: DCISMI, Ductal carcinoma in situ with microinvasive; OR, odds ratio; CI; confidence interval; DCE-MRI, dynamic contrast enhanced MRI.

# a, b, c, d and e represent phases 1, 2, 3, 4 to 5 of dynamic enhancement, respectively.
